# Career Success Criteria Clarity as a Predictor of Employment Outcomes

**DOI:** 10.3389/fpsyg.2020.00540

**Published:** 2020-04-16

**Authors:** Lu Xin, Wenxia Zhou, Mengyi Li, Fangcheng Tang

**Affiliations:** ^1^College of Economics and Management, Beijing University of Chemical Technology, Beijing, China; ^2^School of Labor and Human Resources, Renmin University of China, Beijing, China

**Keywords:** career success, career satisfaction, person–job fit, well-being, career decision-making self-efficacy

## Abstract

Drawing on the goal-setting theory and social cognitive career theory (SCCT), this study empirically proposes an operational definition of career success criteria clarity (CSCC) and further explores its impact on career satisfaction, person–job fit, and subjective well-being through the mediating role of career decision-making self-efficacy (CDSE). A pilot study of 231 samples showed that the CSCC scale had good reliability and validity. To further test the effects of CSCC on crucial employment outcomes, as well as the mediating role of CDSE, 240 employees were included in an additional survey. Structural equation modeling path analysis supported all the expected hypotheses. Results indicated that: (1) CSCC was positively correlated to career satisfaction; (2) CSCC was positively correlated to person–job fit; (3) CSCC was positively correlated to subjective well-being; (4) CSCC was positively correlated to CDSE; (5) CDSE fully mediated the relationship between CSCC and career satisfaction; (6) CDSE fully mediated the relationship between CSCC and person–job fit; and (7) CDSE partly mediated the relationship between CSCC and subjective well-being. The results contributed to social cognitive career theory model and provided suggestions for both the career educators and consultants.

## Introduction

The careers landscape has changed remarkably over the last few decades due to fast-changing employment patterns. Previously, most people developed linear careers within one organization with relatively aligned interests and targets, such as pay raise and promotions, which were considered as objective career success criteria (Stumpf, 2014). Nowadays, the concepts of boundaryless career and protean career have been advanced by the fact that employees play an increasing active role in career development and move voluntarily across organizational boundaries for better employability and career success (Arthur, 1994; Hall, 2002). People pursue jobs that are meaningful to them personally and assess career success more subjectively based on their own standards, needs, values, and aspirations (Arthur and Rousseau, 1996).

As attention turned to subjective career success, Hall and Chandler (2005) reminded scholars to avoid “either-or” discourse: both objective standards and subjective feelings should be considered while assessing career success. Numerous qualitative studies have provided evidence on the multidimensionality of career success. For instance, Gattiker and Larwood (1986) demonstrated that career success encompassed job success, personal success, financial success, hierarchical success, and life success. Moreover, Dries et al. (2008) developed a multidimensional model for career success and concluded nine dimensions: performance, advancement, self-development, creativity, security, satisfaction, recognition, cooperation, and contribution. One fact that cannot be ignored is the overlap between objective career success and subjective career success. For instance, income and social status are objective factors in career success, but further assessment of career success is based on subjective insights. Therefore, when discussing the criteria of career success, more attention should be paid to diverse views and personal insights, rather than simply applying universal standards (Mayrhofer et al., 2016).

Combined with qualitative and quantitative methods, Zhou et al. (2013) developed a three-dimensional framework for career success criteria, including fulfillment of intrinsic psychological needs, balance between work and nonwork lives, and extrinsic rewards. Scholars conducted further studies to explore the antecedents and outcomes of career success criteria (e.g., Pan and Zhou, 2013; Zhou et al., 2016). Since career success criteria denote perception, cognition, value, and self-defined goals of one’s career, people have different priorities and vary in the degree of recognition of each dimension (Zhou et al., 2016). With different criteria, people will behave differently and choose different career paths. For example, some people work overtime for higher payment; someone earns less to take good care of his or her children; while others build social networks, seeking opportunities for promotion.

There are no right or wrong paths, and all paths lead to success of different sorts. Scholars have offered academic evidence, such as Dyke and Murphy (2006) and Visagie and Koekemoer (2014), who found that individuals with successful careers had distinct success criteria. This means that the content of the criteria does not determine one’s career success. However, according to Mayrhofer et al. (2016), career success criteria guide and motivate individuals to develop their careers. Therefore, a crucial question of how career success criteria affect career development has arisen.

From the perspective of the goal-setting theory, the degree of certainty or clarity is a significant concomitant of a goal, determining goal achievement (Locke, 1967). Given that career success criteria denote employees’ ultimate career goals (Arthur et al., 2005; Heslin, 2005), this study proposes the concept of career success criteria clarity (CSCC) and attempts to demonstrate the role of such a concept in career development through rigorous empirical testing. It contributes to the existing literature on career success and career development in at least three ways. First, while previous research focused on the content of career success criteria, this research pays more attention to the clarity of career success criteria and provides a new perspective to advance the current understanding in the field of career success. Second, based on the goal-setting theory, it examines the predictive validity of CSCC on crucial career-related outcomes, enriching the literature of career development. Third, this research attempts to deepen the understanding of career decision-making self-efficacy (CDSE) by testing its intermediary role in the relationship between CSCC and employment outcomes to complete the social cognitive career theory (SCCT) models.

## Career Success Criteria Clarity and Its Distinctiveness From Related Career Concepts

The relationships between career success criteria and career development outcomes are complicated and have not been well explained (Sortheix et al., 2015). According to the goal-setting theory, vague goals, such as “do one’s best,” could not lead to the best performance due to an almost complete lack of motivation effect on employees. On the contrary, such a vague goal offers an excuse for those with low performance. Therefore, the clarity of the expected achievement is important (Locke and Latham, 2006), and we assume that CSCC has a crucial impact on career development.

Career success criteria clarity is based on the concept of career success criteria, which are components of the dynamic self-system, a constantly changing combination of self-schemas that represent one’s attitude, belief, value, and goal in career development and further guide his or her emotions, information processing and vocational behaviors (Hoyle and Sherrill, 2006; Zhou et al., 2013). While developing careers, people continuously construct their career success criteria (Dries et al., 2008). The better one’s career success criteria are constructed, the better clarity the person has for career success criteria. Approximately 30 years ago, Campbell (1990) introduced a new concept in psychology, called self-concept clarity (Campbell, 1990; Campbell and Lavallee, 1993; Campbell et al., 1996). As a structural aspect of self-concept, it refers to the extent to which an individual’s self-concept is clearly and confidently defined. The degree of clarity reflects how well the components of self-concept are organized. Similar to self-concept, career success criteria are also viewed as a cognitive schema based on personal values, traits, and self-relevant information (Kihlstrom and Cantor, 1984; Zhou et al., 2013). Hence, we argue that clarity is a key characteristic that is likely to make career success criteria effective in prompting proactive career behaviors. The clarity develops over time as individuals think about goals and aspirations for their careers, observe role models, and consider what they value most in their careers, implying a construction process. We thus propose that the concept of CSCC offers a new perspective for career success studies and advances the current understanding on career success criteria by distinguishing the structure from the contents.

In addition, relevant studies increasingly reveal the importance of clarity in career domain, such as future work self-salience (Strauss et al., 2012; Cai et al., 2015; Taber and Blankemeyer, 2015). Future work self refers to the image of individuals’ ideal future working life, while future work self-salience indicates the degree to which the future work self is clear and easy to image (Strauss et al., 2012). Although focusing on a different facet of a career, a point in common between future work self-salience and CSCC is that a clearer career cognition and salient vocational goals can lead to better career development. Both future work self-salience and CSCC can motivate vocational behaviors and generate strategies to keep striving toward goals. Future work self-salience is usually measured by a single-dimension scale developed by Strauss et al. (2012). A sample item is “The mental picture of this future is very clear.” People vary in generating mental pictures for future working lives. Some may develop general images, such as being an expert on chemistry, while others may have specific images, including detailed career position, job content, and salary level, resulting in high subjectivity. CSCC is distinct from future work self-salience in that career success criteria have a multidimensional structure, which provides a more comprehensive perspective on clarity.

For the reasons mentioned above, the current research focuses on the clarity of career success criteria, which is a structural aspect of career success criteria and is defined as the extent to which the criteria of assessing career success is clearly and confidently constructed. Accordingly, the measurement of CSCC was developed on the basis of the career success criteria scale. The reliability and validity of the measurement were tested with a pilot study which has been published on a Chinese journal with the whole process of developing the scale. The results will be introduced in brief for a better understanding of the current research and to provide an English version of the scale for international scholars. For more details, please refer to Xin et al. (2019). In addition, the main study aims to examine the predictive validity of CSCC on crucial outcomes with an integrated conceptual framework. To be specific, current research has two goals: (1) testing whether CSCC contributes to career satisfaction, person–job fit, and subjective well-being; and (2) examining the mediation effect of CDSE. Overall, two consecutive studies were conducted: the first one examining the validity and reliability of the scale of CSCC and the second study explaining the influences of CSCC on both vocational and life outcomes.

## Pilot Study – Measuring Career Success Criteria Clarity

The pilot study was conducted to develop a measure for CSCC and to test the quality of the items, as well as reliability and validity. Data were collected from three universities in Beijing, China. Permission to conduct this study was obtained from the university ethics boards. The students involved in this research were informed that their personal information would be kept confidential and that all the data would be used only for research purposes. In total, we distributed 300 surveys and received 231 (77%) responses. The mean age of the participants was 22 years (SD = 0.96); 6.8% of the participants were freshmen, 20.3% were sophomores, 51.7% were juniors, 16.9% were seniors, and 4.4% were graduates.

The measure of CSCC considers the individuals’ judgment of clarity based on the short version of the career success criteria scale (Pan and Zhou, 2015). As a result, we modified the items measuring career success criteria to measure CSCC. We asked the participants to rate the extent to which they were clear and confident when judging each item of the scale of career success criteria. For example, one of the items measuring career success criteria is “One has achieved power over an organization.” We modified this item into “I am clear and confident regarding my views on whether achieving power over an organization represents career success.” Students responded on a 7-point Likert scale ranging from 1 (strongly disagree) to 7 (strongly agree).

As shown in Table 1, 10 items of CSCC all met the standards of CR, CITC, and exploratory factor analysis (EFA) testing, indicating good quality of those items. In addition, our scale had high reliability (Cronbach’s α = 0.94) and high discriminant validity (AVE = 0.59).

**TABLE 1:** CR, CITC, EFA outcome of career success criteria clarity’s items (*N* = 231).

Career success criteria clarity			
			Factor
Items	CR sig.	CITC	loadings
Whether being continuously promoted to higher level in an organization represents career success	0.00	0.64	0.70
Whether achieving power over an organization represents career success	0.00	0.70	0.76
Whether making much money through work represents career success	0.00	0.75	0.81
Whether one’s talents and potential being fully utilized in career represents career success	0.00	0.84	0.88
Whether being happy during work represents career success	0.00	0.82	0.86
Whether being continuously engaged in challenging work represents career success	0.00	0.82	0.86
Whether feeling fulfilled at work represents career success	0.00	0.78	0.83
Whether enjoying life in career represents career success	0.00	0.76	0.81
Whether achieving balance between life and work represents career success	0.00	0.72	0.78
Whether maintaining good physical and mental health represents career success	0.00	0.63	0.70
Eigen value		6.39	
Cumulative explaining variation (%)		63.93	

*CR, critical ratio; CITC, corrected item-total correlation; EFA, exploratory factor analysis.*

## Main Study – Consequences of Career Success Criteria Clarity

The SCCT views value orientations as factors that affect one’s career expectations and decisions (Lent et al., 2000). The goal-setting theory also demonstrates that the clarity of goals has direct impact on goal achievement (Locke, 1967). Since career success criteria reflect career-relevant values and denote the ultimate career goals that individuals pursue, we propose that CSCC can positively predict career success and expectations.

Of the 216 research articles on subjective career success in 30 years, 86% of them used career satisfaction to measure subjective career success (Ng and Feldman, 2014). Career satisfaction has also been seen as a significant measure of a career as a whole (Gattiker and Larwood, 1988; Gattiker and Larwood, 1989). To be coherent with previous research, we choose career satisfaction as an outcome of CSCC. In the era of boundaryless careers, person–job fit is considered to be a wise choice during extensive interorganizational mobility (Tinsley, 2000), so the effect of CSCC on person–job fit is also tested in this paper. Moreover, the purpose of a career for most people is to pursue a good life, which means a flourishing life with high-level well-being (Huppert, 2009; Coffey et al., 2016). Therefore, the influence of career success criteria on well-being is also examined in this study.

Overall, the aim of this study is to examine the impact of CSCC on career satisfaction, person–job fit, and subjective well-being. Since individuals with clear career success criteria will have more confidence in making career decisions and in turn achieve career success and life goals, we will also examine the mediation role of CDSE in the relationships mentioned above (Dietrich et al., 2013).

### Theoretical Background and Hypotheses Development

#### Career Success Criteria Clarity and Career Satisfaction

Career satisfaction refers to an individual’s overall judgment and evaluation of personal career development (Greenhaus et al., 1990). Scholars conducted numerous studies to identify the predictors of career satisfaction, including personal, vocational, and organizational factors, such as gender, personality, and organizational climate (Frank et al., 1999; El Baroudi et al., 2017). Judge et al. (2010) also noticed that a high level of core self-evaluation could motivate employees to shoulder more responsibility and achieve improved performance, in turn leading to heightened career satisfaction. Similar to core self-evaluation, CSCC represents an individual’s self-cognition and values in a career. A high degree of CSCC would motivate employees to make progress in career development, such as participating in challenging or extra tasks, which are beneficial to achieving career goals, resulting in high rewards and career satisfaction.

According to the goal-setting theory, the clarity of goals has a direct impact on goal achievement (Locke, 1967). When individuals have more clarity on career success criteria, they tend to have a stronger perception of their current career development and pursue career success proactively, and in turn gain career satisfaction. Therefore, as a personal attribute for careers, we expect CSCC would influence career satisfaction. Consequently, we propose the following hypothesis:

H1: CSCC is positively related to career satisfaction.

#### Career Success Criteria Clarity and Person–Job Fit

Person–job fit is defined as the consistency or the degree of matching between an individual’s characteristics and those of the job or tasks that are performed at work (Lee et al., 2010). In the past, most people developed their careers within one organization, so they took organizations into account foremost when making career choices. However, with the advent of boundaryless careers, people now pay more attention to their personal requirements and job features, such as the salary package, job duties, and promotion opportunities. Thus, person–job fit is considered to be a wise career choice (Tinsley, 2000). Many studies indicate that person–job fit should take two aspects into consideration. On the one hand, specific knowledge and employee skills should be matched with their job requirements, namely, demands–abilities fit. On the other hand, employees’ needs and desires should fit with their jobs, namely, needs–supplies fit (Ardıç et al., 2016). High-level person–job fit means that the job satisfies the employee’s needs, capacities, and values, which further contributes to his or her career development.

Previous research has revealed the positive effects of person–job fit on organizational citizenship behavior and job performance and the negative impact on turnover intention (Hassan et al., 2012; Lin et al., 2014). Therefore, person–job fit is an essential goal in job searching and occupational mobility. According to the goal-setting theory, individuals tend to realize their aspirations more easily when their visions are clearer (Locke et al., 1968). Clear career success criteria are motivational resources because they support the process through which vocational goals are clearly defined and strategies are generated for achieving these goals (Strauss et al., 2012). Individuals with a high level of CSCC tend to have a better understanding of what skills should be equipped and reduce role ambiguity, improving demand–ability fit (Cai et al., 2018). They will also have a better understanding of what they want from their jobs, promoting need–supply fit (Dietrich et al., 2013). In this case, they are more capable to find suitable jobs. Consequently, we propose the following hypothesis:

H2: CSCC is positively related to person–job fit.

#### Career Success Criteria Clarity and Well-Being

How to gain well-being through career development is becoming an interest of researchers and practitioners. Some believe that one way to achieve well-being is to strive for career success (Diener and Seligman, 2004). However, the relationship between career success and well-being is complicated, a position supported by both practical and academic evidence. For one thing, high income and high status-position with power may increase personal well-being. For another, time constraints, stress, and other costs may impair life satisfaction. The famous paradox of happiness stems from the discussion of the relationship between income and well-being (Easterlin, 2001).

Apart from inconsistent results, most scholars focus on the relationship between career outcomes and well-being, neglecting the influence of vocational cognition and values on happiness. Career success criteria not only denote ultimate career values and goals that individuals pursue but also reflect values and goals of one’s own view of life. For example, the work–life balance dimension of career success criteria expresses the goal of acquiring balance between work and nonwork life. The fundamental reason is that career and life are inalienable, as career is a process through which people endow meanings to their vocations (Savickas, 2013). As a result, it is integral to discuss the impact of CSCC on both career and life outcomes.

Moreover, drawing upon the goal-setting theory, goals are easier to achieve when they are set more clearly (Locke, 1967). When individuals have a clear picture of what they are pursuing for career success and their whole lives, they will endeavor to achieve their goals by improving work-related abilities and advancing career plans accordingly. This process generates a sense of control and security over a career, enhancing their overall sense of well-being. A high level of CSCC, representing one’s self-confidence and clarity regarding career success, can regulate employees’ psychological process and reduce their negative feelings, further ensuring their subjective well-being (Liu et al., 2016). Consequently, we propose the following hypothesis:

H3: CSCC is positively related to well-being.

#### The Mediation Role of Career Decision-Making Self-Efficacy

Lent et al. (1994, 2000) have developed the SCCT to offer a unifying framework for explaining how people generate vocational interests, make relevant choices, and pursue positive vocational outcomes. It posits that personal traits primarily affect individuals’ self-efficacy beliefs and further contribute to career development and vocational performance (Brown et al., 2011). As a crucial component in the SCCT model, self-efficacy represents an individual’s confidence in his or her abilities to accomplish tasks (Hackett and Betz, 1981). In the field of career development, CDSE refers to one’s belief that he or she can successfully complete the tasks which are necessary for making career decisions, compassing five dimensions: self-appraisal, occupational information, goal selection, planning, and problem solving (Betz et al., 1996). Many studies have demonstrated that CDSE serves as an important indicator of individuals’ vocational behaviors and outcomes, such as job satisfaction (Caprara et al., 2003; Peng and Mao, 2015; Li et al., 2017), intrinsic satisfaction (Borgogni et al., 2013), and career choice commitment (Jin et al., 2009). According to SCCT, career-related expectations and decisions are influenced by personal and contextual factors (Brown et al., 2011). Personality and other social variables (e.g., race, gender, certainty, extraversion, conscientiousness, and cultural mistrust) have been shown to have significant correlations with CDSE (Bullock-Yowell et al., 2011). Representing clear vocational goals and relevant cognition, CSCC enables individuals to generate more accurate self-appraisal and gain occupational information more effectively. They will be confident to make career decisions and prepare for the future. Thus, we suppose that CSCC would contribute to a high level of CDSE. Moreover, individuals with a high level of self-efficacy are motivated to strive for their goals (Bandura, 1977). They are inclined to adopt a problem-focused coping style and seek for external resources like social support (Chang and Edwards, 2015). They are more capable of dealing with challenges and solving difficult problems (Liu et al., 2016), ensuring a high level of career satisfaction, person–job fit, and subjective well-being. Therefore, our study sought to examine the mediating effect of CDSE in the relationships between CSCC and career satisfaction, person–job fit, and well-being. Consequently, we propose the following hypotheses:

H4: CDSE mediates the relationship between CSCC and career satisfaction.

H5: CDSE mediates the relationship between CSCC and person–job fit.

H6: CDSE mediates the relationship between CSCC and well-being.

**FIGURE 1:**
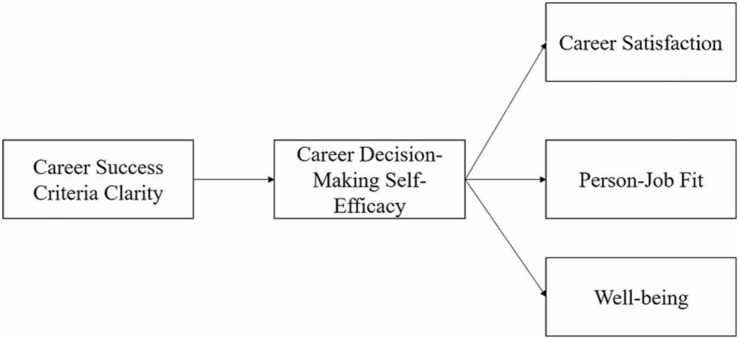
Research model.

Combining all the hypotheses, we propose a mediation model to test the relationships between CSCC and career satisfaction, person–job fit, and well-being with CDSE as a mediator. Figure 1 shows the research model.

### Samples and Procedures

Since the early stages of a career are critical for the formation of career values and cognition, the rapid change of employment models brings more challenges and difficulties to young people as they establish careers (Sortheix et al., 2015). Therefore, we particularly focus on employees in their early career stages in this research. The data come from the on-job graduate students of a university in Beijing and previous graduates of a college in Taiyuan, China. We distributed questionnaires *via* e-mail, and a total of 240 usable sets of questionnaires were obtained out of 500, yielding an overall response rate of 48%. All the participants held a bachelor’s degree. Part of them began to work with no further education after undergraduate period. Others were on-the-job graduates. Among the participants, 80.40% were women, and the average age of the respondents was 26.92 years (SD = 3.92).

### Measures

The scales used in this study were developed originally in English. Following recommended procedures (Brislin, 1980), we translated them into Chinese. Then, we back translated the Chinese versions of those scales into English. We invited two doctoral students majoring in English to compare original versions with the back translations. According to their suggestions, we modified a few items to ensure accuracy and clarity.

#### Career Success Criteria

Career success criteria were measured with the short version of 10 items developed by Pan and Zhou (2015). This scale was used in previous studies and had good validity (Zhou et al., 2016). Participants were asked to rate the extent to which they agree is appropriate to assess career success as a criterion on a 7-point Likert scale ranging from 1 (strongly disagree) to 7 (strongly agree). For instance, “Career success means that one’s talents and potential capacities are fully utilized in his or her career.” Cronbach’s alpha for the scale was 0.90.

#### Career Success Criteria Clarity

To assess CSCC, we asked the participants to rate the extent to which they were clear and confident while making judgment on each item of the scale of career success criteria. A sample item was “I am clear and confident regarding my views on whether achieving power over an organization represents career success.” Students responded on a 7-point Likert scale ranging from 1 (strongly disagree) to 7 (strongly agree). Cronbach’s alpha for the scale was 0.94.

#### Career Decision-Making Self-Efficacy

To assess self-efficacy in career decision-making, the scale of 25 items developed by Betz et al. (1996) was used. This scale was used in previous research and had good validity (Chung, 2002; Zhou et al., 2016). A sample item was “Choose a major or career that will fit your interests.” Participants rated their confidence on decision-making tasks on a 7-point Likert scale ranging from 1 (not confident at all) to 7 (extremely confident). Cronbach’s alpha for the scale was 0.97.

#### Career Satisfaction

Career satisfaction was measured with a widely used scale developed by Greenhaus et al. (1990). This scale was validated by previous studies (Seibert et al., 1999). A sample item was “I am satisfied with the success I have achieved in my career.” Participants responded to five items on a 7-point Likert scale ranging from 1 (strongly disagree) to 5 (strongly agree). Cronbach’s alpha for the scale was 0.92.

#### Person–Job Fit

We used the four-item scale adopted from Saks and Ashforth (1997) to measure the level of fit between employee and his or her current work. This scale was validated by previous studies (Carless, 2005). An example of the items used was “To what extent do your knowledge, skills and abilities match the requirements of the job.” The Cronbach’s alpha for this scale was 0.87.

#### Well-Being

In this study, we use the positive emotion subscale of PANAS (Positive and Negative Affect Schedule; Watson et al., 1988) to measure well-being following previous studies (Pan and Zhou, 2015; Pan et al., 2016). This scale contains 10 items assessing positive affect throughout the past few weeks, involving attentive, active, alert, determined, excited, enthusiastic, inspired, proud, interested, and strong. Each item was rated on a 5-point Likert scale ranging from 1 (very slightly or not at all) to 5 (extremely). This scale was validated by previous studies (Pan and Zhou, 2015; Pan et al., 2016). The Cronbach’s alpha in this study was 0.85.

#### Control Variables

We controlled for the effects of gender and age. On the basis of previous studies, frequently used control variables in research concerning to career success, well-being, and career satisfaction included age, gender, educational background, and career stages (Judge and Bretz, 1994; Stumpf, 2014; Zhou et al., 2016; Batz and Tay, 2018). This study focused on employees in their early careers, and all the participants graduated with a bachelor’s degree and no subsequent graduate degree. As a result, only gender and age were controlled in this study.

## Results

### Common Method Bias Test

This study adopted a self-report method to collect data. Therefore, we used Harman’s single-factor test to address the issue of common method bias according to Podsakoff and Organ (1986). We loaded all the items into an exploratory factor test and examined the unrotated factor solution (Podsakoff et al., 2003). The result of exploratory factor analysis in this study showed that the first factor accounts for 30.59% of total variance. No one factor accounted for the majority of the covariance among the measures. Therefore, common method bias was not a problem in this study.

To further validate this result, we also applied confirmatory factor test following previous studies (e.g., Korsgaard and Roberson, 1995; Iverson and Maguire, 2000). As shown in Table 2, single-factor model indices were worse than those of the six-factor model, supporting the result of exploratory factor test.

**TABLE 2:** Model comparison.

Model	*χ*^2^	*df*	*χ*^2^*/df*	RMSEA	CFI	TLI	*Δχ*^2^	*Δdf*
Six-factor Model	976.22	480	2.03	0.07	0.93	0.92		
Five-factor Model	1, 101.20	485	2.27	0.07	0.91	0.90	124.98***	5
Four-factor Model-1	1, 537.74	489	3.15	0.10	0.85	0.84	561.52***	9
Four-factor Model-2	1, 467.54	489	3.00	0.09	0.86	0.85	491.32***	9
Three-factor Model	1, 894.56	492	3.85	0.11	0.80	0.78	918.33***	12
Two-factor Model	3, 166.29	494	6.41	0.15	0.62	0.59	2,190.07***	14
Single-factor Model	3, 596.34	495	7.27	0.16	0.55	0.52	2,620.12***	15

***p < 0.001. Five-factor Model: Career success criteria clarity (CSCC) + Career success criteria, Career decision-making self-efficacy (CDSE), Career satisfaction, Person–job fit, Well-being. Four-factor Model 1: CSCC + Career success criteria, CDSE + Career satisfaction, Person–job fit, Well-being. Four-factor Model 2: CSCC + Career success criteria, CDSE, Person–job fit, Well-being + Career satisfaction. Three-factor Model: CSCC + Career success criteria, CDSE + Career satisfaction + Well-being, Person–job fit. Two-factor Model: CSCC + Career success criteria + CDSE + Career satisfaction + Well-being, Person–job fit. Single-factor Model: CSCC + Career success criteria + CDSE + Career satisfaction + Well-being + Person–job fit. CFI, Comparative Fit Index; RMSEA, root mean square error of approximation; TLI, Tucker–Lewis Index.

### The Measurement Model

In this study, we used confirmatory factor analysis (CFA) to test the discriminant validity of the measurement model. KMO test and the Bartlett’s test of sphericity were conducted before CFA. The KMO value was 0.82, and the result of Bartlett’s test sphericity was significant (*p* < 0.001). Therefore, this sample was suitable for factor analysis (Hair et al., 1995).

We used several fit indices to evaluate the fitness of the model according to established practice (Hu and Bentler, 1999; Byrne, 2013), including χ^2^/df, Comparative Fit Index (CFI), Tucker–Lewis Index (TLI), and root mean square error of approximation (RMSEA). The results of the CFA were shown in Table 2. The measurement model fit with the data best than other models with χ^2^/ df = 2.03, CFI = 0.93, TLI = 0.92, and RMSEA = 0.07 (Table 2). Distinctiveness of the measurement model in this study was ensured.

### Descriptive Statistics

As shown in Table 3, career satisfaction is positively related to CSCC (*r* = 0.44, *p* < 0.01), supporting Hypothesis 1. Person–job fit is positively correlated to CSCC (*r* = 0.34, *p* < 0.01), supporting Hypothesis 2. Well-being is positively related to CSCC (*r* = 0.34, *p* < 0.01), supporting Hypothesis 3. CDSE is positively related to CSCC (*r* = 0.48, *p* < 0.01).

**TABLE 3:** Means, standard deviation, and correlations for variables (*N* = 240).

Variables	Means	SD	1	2	3	4	5	6	7
1. Gender	1.82	0.38	–						
2. Age	26.92	3.92	0.08	–					
3. Career success criteria clarity	4.84	1.04	0.07	0.01	–				
4. Career decision-making self-efficacy	4.81	1.13	0.03	0.00	0.48**	–			
5. Career satisfaction	4.72	1.30	0.04	0.04	0.44**	0.72**	–		
6. Person–job fit	3.66	0.81	0.07	0.02	0.34**	0.69**	0.63**	–	
7. Well-being	4.58	1.20	−0.07	0.02	0.34**	0.39**	0.36**	0.37**	–

**p < 0.01.

### The Structural Model

**FIGURE 2:**
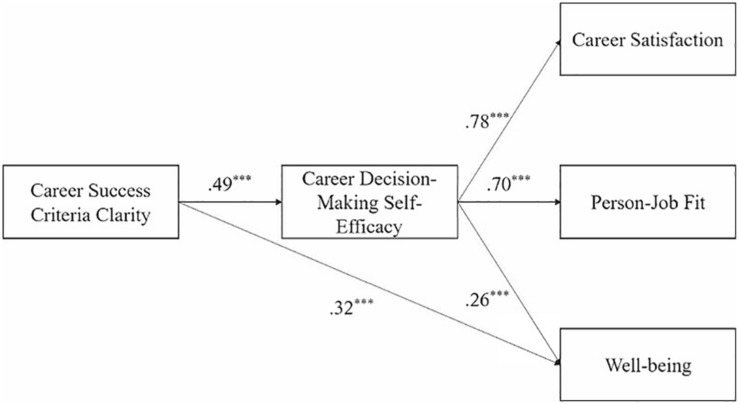
Result of research model test.

Since our model includes multiple dependent variables and mediator, we used structural equation modeling to analyze the whole model simultaneously. As shown in Table 4, M3 is significantly better than other models. Results are shown in Figure 2, which support Hypothesis 4, Hypothesis 5, and Hypothesis 6.

**TABLE 4:** Structural equation modeling comparison.

Model	*χ*^2^	*df*	*χ*^2^*/df*	RMSEA	CFI	TLI	*Δχ*^2^	*Δdf*
M0	731.80	395	1.85	0.06	0.93	0.93		
M1	731.64	394	1.86	0.06	0.93	0.93	0.16	1
M2	731.69	394	1.86	0.06	0.93	0.92	0.11	1
M3	716.80	394	1.82	0.06	0.93	0.93	15.00***	1

***p < 0.001. M1 adds a direct path from career success criteria clarity (CSCC) to career satisfaction based on M0; M2 adds a direct path from CSCC to person–job fit based on M0; M3 adds a direct path from CSCC to well-being based on M0. CFI, Comparative Fit Index; RMSEA, root mean square error of approximation; TLI, Tucker–Lewis Index.

### Bootstrapping Results of Mediation Effects

To further confirm the mediation effects of CDSE, this study adopted the biased-corrected bootstrapping method developed by Preacher and Hayes (2008). Table 5 showed the results of mediation model, and Table 6 showed the result of indirect effects. In Table 5, equation 1 showed the results of the total effect model. Equations 2 and 3 showed the results of mediation model.

**TABLE 5:** Results of mediation model (*N* = 240).

Dependent variables	Variables	Equation 1 Total effect			Equation 2 OV: CDSE			Equation 3 OV: CS/PJF/WB		
		*β*	*SE*	*t*	*B*	*SE*	*t*	*β*	*SE*	*t*
Career satisfaction	Gender	0.03	0.20	0.16	0.03	0.17	0.20	0.01	0.15	0.04
	Age	0.04	0.08	0.46	–0.02	0.07	–0.30	0.06	0.06	0.90
	CSCC	0.49	0.08	6.08***	0.49	0.07	7.46***	0.08	0.07	1.25
	CDSE							0.83	0.06	13.26***
	*R*^2^	0.15			0.21			0.54		
	*F*	12.74***			19.06***			61.46***		
Person–job fit	Gender	0.13	0.13	1.01	0.06	0.17	0.33	0.11	0.11	1.01
	Age	0.00	0.05	0.07	–0.01	0.07	–0.21	0.01	0.04	0.25
	CSCC	0.25	0.05	4.79***	0.48	0.07	7.35***	0.02	0.05	0.36
	CDSE							0.47	0.04	11.08***
	*R*^2^	0.11			0.21			0.44		
	*F*	8.67***			18.70***			40.94***		
Well-being	Gender	–0.37	0.20	–1.88	0.03	0.17	0.17	–0.38	0.19	−1.97*
	Age	0.02	0.08	0.24	–0.01	0.07	–0.19	0.02	0.08	0.29
	CSCC	0.49	0.08	6.38***	0.49	0.07	7.47***	0.35	0.08	4.17***
	CDSE							0.29	0.08	3.63***
	*R*^2^	0.17			0.21			0.22		
	*F*	13.91***			19.08***			14.32***		

*p < 0.05, ***p < 0.001. CDSE, career decision-making self-efficacy; CS, career satisfaction; CSCC, career success criteria clarity; OV, outcome variable; PJF, person–job fit; WB, well-being.

**TABLE 6:** Results of indirect effect (*N* = 240).

Dependent variables	*β*	SE	LLCI	ULCI
Career satisfaction	0.40	0.07	0.27	0.53
Person–job fit	0.23	0.04	0.15	0.32
Well-being	0.14	0.07	0.03	0.30

*LLCI, lower limit of the CI; ULCI, upper limit of the CI.*

As the result of equation 1 showed, CSCC had a significantly positive effect on career satisfaction (*β* = 0.49, *p* < 0.001), person–job fit (*β* = 0.25, *p* < 0.001), and well-being (*β* = 0.49, *p* < 0.001). These results provided further support for Hypotheses 1, 2, and 3. In equation 2, the positive effects of CSCC on CDSE were all significant (career satisfaction: *β* = 0.49, *p* < 0.001; person–job fit: *β* = 0.48, *p* < 0.001; well-being: *β* = 0.49, *p* < 0.001). In equation 3, only CDSE positively predicted career satisfaction (*β* = 0.83, *p* < 0.001) and person–job fit (*β* = 0.47, *p* < 0.001), while both CSCC and CDSE positively predicted well-being (*β* = 0.35, *p* < 0.001; *β* = 0.29, *p* < 0.001, respectively), illustrating that: (1) CDSE fully mediated the relationship between CSCC and career satisfaction, (2) CDSE fully mediated the relationship between CSCC and person–job fit, and (3) CDSE partly mediated the relationship between CSCC and well-being. In Table 6, 95% confidence interval of CDSE on these three dependent variables did not include 0. Therefore, Hypotheses 4, 5, and 6 were further supported.

## Discussion

This study proposes a new concept of CSCC, enriching the literature of career success. Previous studies have mainly focused on the content of career success criteria, while our study explores the structural aspect of career success criteria from a constructive perspective. The development of CSCC scale provides an instrument for future studies and offers an evaluation tool for individuals and organizations to diagnose career obstacles and manage career development.

Drawing on the goal-setting theory, we construct a conceptual model to test the predictive effect of CSCC on career satisfaction, person–job fit, and well-being through the mediation role of CDSE. For careers, our study provides empirical evidence for the positive effect of CSCC on career satisfaction and person–job fit. Although numerous studies have explored the antecedents of career satisfaction, most of them emphasized the internal attributes of career management, ignoring the proactivity of individuals (Magee, 2013). Since CSCC is the degree to which individuals construct their career success criteria, this indicator reflects individuals’ proactivity and contributes to career satisfaction studies. Besides, the significant predictive role of CSCC on person–job fit has also been well examined in this study. People with better understanding of what they want from their jobs are more capable of finding suitable jobs.

Apart from vocational outcomes, CSCC was also proved to be a fundamental explanation of well-being. Diener and Seligman (2004) point out that career success is an important source of well-being, and many studies discuss the relationship between career success and well-being. However, they focus more on the outcomes or feelings of career success, ignoring the cognition and values underlying career success. Our study reinforces the significance of vocational cognition and values in generating overall well-being. In general, the results illustrate that CSCC influences not only vocational outcomes but also overall well-being.

This study also reveals the underlying mechanism between CSCC and the outcomes above by examining the mediation role of CDSE, expanding the SCCT model (Lent et al., 2000). Previous research shows that personality and some social variables have significant correlations with CDSE (Bullock-Yowell et al., 2011). This study contributes to academic literature by proving that individuals with clear vocational goals and relevant cognition could generate more accurate self-appraisals and gain occupational information more effectively. Consequently, they will be more confident to make career choices and therefore gain positive outcomes.

Most of the studies regarding decision-making self-efficacy take SCCT (Lent et al., 1994; Lent et al., 2000) as their theoretical basis (e.g., Gushue and Whitson, 2006). However, we also use the goal-setting theory to explain the relationships mentioned above in our study. Locke and Latham (2006) believe that “the success of goal setting depends upon taking account of the mediators and moderators that determine its efficacy and applicability” (p. 268). In response to their call, this study examined the mediating effect of CDSE. Moreover, this research is in line with Locke and Latham (2006) in exploring the linkage between goals and cognition, namely, the relationship between career satisfaction, person–job fit, well-being, and CSCC.

This study offers crucial practical implications for both employees and organizations. First, the measurement we developed in this study could be adopted by employees to identify their priorities and career goals, aiding in the choice of appropriate strategies in career self-management. Meanwhile, organizations can also gain a better understanding of employees with this instrument and provide effective incentive and career development planning. Second, as CSCC positively affects career-related outcomes and subjective well-being, career educators and counselors should attend to the clarity of career success criteria when designing programs or interventions for employees, especially the job market entrants. They could encourage individuals to think about goals and aspirations for their career as well as providing role models to guide them to improve their clarity in career success criteria. As a result, employees can achieve better employment status and gain well-being on the one hand and organizations can retain the talents more efficiently by increasing their career satisfaction on the other. Moreover, as CSCC plays a foundational role in career development by promoting CDSE, career educators and counselors should also be aware that developing individuals’ career success criteria is an effective way to facilitate the career decision-making process and further leads to superior employment outcomes.

This study has several limitations. First, since the design of this study cannot support any causal conclusions for the relationships between these variables, further research should adopt a more rigorous design, such as a longitudinal design, to test causal effects. Second, as the mediation model revealed in this study was based on a sample of employees at an early career stage, whether this model can be supported with samples of employees in middle to late career stages awaits future examinations. Additionally, whether further extension of this model to other outcomes remains to be discovered. Furthermore, we advocate for further research to discover moderators that would strengthen the effects of CSCC on employment outcomes.

Overall, we believe that the construction of career success criteria is crucial due to representation of the cognition and values in career goals, which results in high career satisfaction, person–job fit, and well-being through CDSE.

## Data Availability Statement

The raw data supporting the conclusions of this article will be made available by the authors, without undue reservation, to any qualified researcher.

## Ethics Statement

An ethics approval was not required according to the guidelines of the Renmin University of China and Beijing University of Chemical Technology as well as national regulations. The principals of the universities supported this research. We sent online questionnaire links to participants. They were informed that their personal information would be kept confidential, and the data would be used only for research purposes. Participants volunteered to take the survey, and they were completely free to quit.

## Author Contributions

In preparing this manuscript, LX and WZ worked together to propose a research topic and develop a theoretical model. LX collected data and drafted the manuscript. WZ and FT participated in the discussion on the research model and helped draft the manuscript. ML improved the *Introduction*, *Hypotheses Development*, and *Discussion* sections. All authors read and approved the final manuscript.

## Conflict of Interest

The authors declare that the research was conducted in the absence of any commercial or financial relationships that could be construed as a potential conflict of interest.

## References

[B1] Ardıç K. Uslu O. Oymak Ö Özsoy E. Özsoy T. (2016). Comparing person organization fit and person job fit. *J. Econ. Manage* 25 5–13. 10.22367/jem.2016.25.01

[B2] Arthur M. B. (1994). The boundaryless career: a new perspective for organizational inquiry. *J. Organ. Behav. Spec. Issue Boundar. Career* 15 295–306. 10.1002/job.4030150402

[B3] Arthur M. B. Khapova S. N. Wilderom C. P. M. (2005). Career success in a boundaryless career world. *J. Organ Behav. Spec. Issue Reconceptual. Career Succ.* 26 177–202. 10.1002/job.290

[B4] Arthur M. B. Rousseau D. M. (1996). *The Boundaryless Career.* New York, NY: Oxford University Press.

[B5] Bandura A. (1977). Self-efficacy: toward a unifying theory of behavioral change. *Psychol. Rev.* 84 191–215. 10.1037/0033-295X.84.2.191 847061

[B6] Batz C. Tay L. (2018). *Gender Differences in Subjective Well-Being. Handbook of Well-Being.* Salt Lake City, UT: DEF.

[B7] Betz N. E. Klein K. L. Taylor K. M. (1996). Evaluation of a short form of the career decision-making self-efficacy scale. *J. Career Assess.* 41 47–57. 10.1177/106907279600400103

[B8] Borgogni L. Russo S. D. Miraglia M. Vecchione M. (2013). The role of self-efficacy and job satisfaction on absences from work. *Eur. Rev. Appl. Psychol.* 63 129–136. 10.1016/j.erap.2012.08.007 25078993

[B9] Brislin R. W. (1980). “Translation and content analysis of oral and written material,” in *Handbook of Cross-Cultural Psychology*, eds Triandis H. C. Berry J. W. (Boston, MA: Allyn & Bacon), 398–444.

[B10] Brown S. D. Lent R. W. Telander K. Tramayne S. (2011). Social Cognitive Career Theory, Conscientiousness, and Work Performance: A Meta-Analytic Path Analysis. *J. Vocat. Behav.* 79 81–90. 10.1016/j.jvb.2010.11.009

[B11] Bullock-Yowell E. Andrews L. Buzzetta M. E. (2011). Explaining career decision-making self-efficacy: personality, cognitions, and cultural mistrust. *Career Dev. Q* 59 400–411. 10.1002/j.2161-0045.2011.tb00967.x

[B12] Byrne B. M. (2013). *Structural Equation Modeling with Mplus: Basic Concepts, Applications, and Programming.* New York, NY: Routledge.

[B13] Cai D. Cai Y. Sun Y. Ma J. (2018). Linking empowering leadership and employee work engagement: The effects of person-job fit, person-group fit, and proactive personality. *Front. Psychol* 9:1304. 10.3389/fpsyg.2018.01304 30108534 PMC6080136

[B14] Cai Z. Guan Y. Li H. Shi W. Guo K. Liu Y. (2015). Self-esteem and proactive personality as predictors of future work self and career adaptability: an examination of mediating and moderating processes. *J. Vocat. Behav.* 86 86–94. 10.1016/j.jvb.2014.10.004

[B15] Campbell J. D. (1990). Self-esteem and clarity of the self-concept. *J. Pers. Soc. Psychol.* 59 538. 10.1037/0022-3514.59.3.538 2231284

[B16] Campbell J. D. Lavallee L. F. (1993). “Who am I? The role of self-concept confusion in understanding the behavior of people with low self-esteem,” in *Plenum series in social/clinical psychology. Self-esteem: The puzzle of low self-regard*, ed. Baumeister R. F. (New York, NY: Plenum Press), 3–20. 10.1007/978-1-4684-8956-9_1

[B17] Campbell J. D. Trapnell P. D. Heine S. J. Katz I. M. Lavallee L. F. Lehman D. R. (1996). Self-concept clarity: measurement, personality correlates, and cultural boundaries. *J. Pers. Soc. Psychol.* 70 141–156. 10.1037/0022-3514.70.1.141

[B18] Caprara G. V. Barbaranelli C. Borgogni L. Steca P. (2003). Efficacy beliefs as determinants of teachers’ job satisfaction. *J. Educ. Psychol.* 95 821 10.1037/0022-0663.95.4.821

[B19] Carless S. A. (2005). Person–job fit versus person–organization fit as predictors of organizational attraction and job acceptance intentions: A longitudinal study. *J. Occup. Organ. Psych.* 78 411–429. 10.1348/096317905x25995

[B20] Chang Y. Edwards J. K. (2015). Examining the relationships among self-efficacy, coping, and job satisfaction using social career cognitive theory: An SEM analysis. *J. Career Assess.* 23 35–47. 10.1177/1069072714523083

[B21] Chung Y. (2002). Career decision-making self-efficacy and career commitment: Gender and ethnic differences among college students. *J. Career Dev.* 28 277–284. 10.1023/A:1015146122546

[B22] Coffey J. K. Wray-Lake L. Mashek D. Branand B. (2016). A multi-study examination of well-being theory in college and community samples. *J. Happiness Stud.* 17 187–211. 10.1007/s10902-014-9590-8

[B23] Diener E. (2000). Subjective well-being: the science of happiness and a proposal for a national index. *Am. Psychol.* 55 34. 10.1037/0003-066X.55.1.34 11392863

[B24] Diener E. Ryan K. (2009). Subjective well-being: A general overview. *S. Afr. J. Psychol.* 39 391–406. 10.1177/008124630903900402

[B25] Diener E. Seligman M. E. (2004). Beyond money: toward an economy of well-being. *Psychol. Sci. Publ. Int.* 5 1–31. 10.1111/j.0963-7214.2004.00501001.x 26158992

[B26] Diener E. Suh E. M. Lucas R. E. Smith H. L. (1999). Subjective well-being: three decades of progress. *Psychol. Bull.* 125 276. 10.1037/0033-2909.125.2.276 7630576

[B27] Dietrich J. Shulman S. Nurmi J. E. (2013). Goal pursuit in young adulthood: the role of personality and motivation in goal appraisal trajectories across 6 years. *J. Res. Pers.* 47 728–737. 10.1016/j.jrp.2013.06.004

[B28] Dries N. Pepermans R. Carlier O. (2008). Career success: constructing a multidimensional model. *J. Vocat. Behav.* 73 254–267. 10.1016/j.jvb.2008.05.005

[B29] Dyke L. S. Murphy S. A. (2006). How we define success: a qualitative study of what matters most to women and men. *Sex Roles* 55 357–372. 10.1007/s11199-006-9091-2

[B30] Easterlin R. A. (2001). Income and happiness: towards a unified theory. *Econ. J.* 11 465–484. 10.1111/1468-0297.00646

[B31] El Baroudi S. Fleisher C. Khapova S. N. Jansen P. Richardson J. (2017). Ambition at work and career satisfaction. *Career Dev. Int.* 22 87–102. 10.1108/cdi-07-2016-0124

[B32] Frank E. Mcmurray J. E. Linzer M. Elon L. (1999). Career satisfaction of us women physicians: results from the women physicians’ health study. *Arch. Intern. Med.* 159 1417. 10.1001/archinte.159.13.1417 10399893

[B33] Fredrickson B. L. Cohn M. A. Coffey K. A. Pek J. Finkel S. M. (2008). Open hearts build lives: positive emotions, induced through loving-kindness meditation, build consequential personal resources. *J. Pers. Soc. Psychol.* 95 1045. 10.1037/a0013262 18954193 PMC3156028

[B34] Gattiker U. E. Larwood L. (1986). Subjective career success: a study of managers and support personnel. *J. Bus. Psychol.* 1 78–94. 10.1007/BF01018805

[B35] Gattiker U. E. Larwood L. (1988). Predictors for managers’ career mobility, success, and satisfaction. *Hum. Relat.* 41 569–591. 10.1177/001872678804100801

[B36] Gattiker U. E. Larwood L. (1989). Career success, mobility and extrinsic satisfaction of corporate managers. *Soc. Sci. J.* 26 75–92. 10.1016/0362-3319(89)90039-6

[B37] Greenhaus J. H. Parasuraman S. Wormley W. M. (1990). Effects of race on organizational experiences, job-performance evaluations and career outcomes. *Acad. Manage. J.* 33 64–86. 10.2307/256352

[B38] Gushue G. V. Whitson M. L. (2006). The relationship among support, ethnic identity, career decision self-efficacy, and outcome expectations in African American high school students: applying social cognitive career theory. *J. Career Dev.* 33 112–124. 10.1177/0894845306293416

[B39] Hackett G. Betz N. E. (1981). A self-efficacy approach to the career development of women. *J. Vocat. Behav*. 18 326–339. 10.1016/0001-8791(81)90019-1

[B40] Hair J. F. Anderson R. E. Tatham R. L. Black W. C. (1995). *Multivariate Data Analysis*, 4th Edn River, NJ: Prentice-Hall, Inc.

[B41] Hall D. T. (2002). *Careers in and out of Organizations.* Thousand Oaks, CA: Sage.

[B42] Hall D. T. Chandler D. E. (2005). Psychological success: when the career is a calling. *J. Organ. Behav.* 26 155–176. 10.1002/job.301

[B43] Hassan M. Akram A. Naz S. (2012). The relationship between person organization fit, person-job-fit and turnover intention in banking sector of Pakistan: The mediating role of psychological climate. *Int. J. Hum. Resour. Stud.* 2 172 10.5296/ijhrs.v2i3.2286

[B44] Heslin P. A. (2005). Conceptualizing and evaluating career success. *J. Organ. Behav.: The International Journal of Industrial, Occupational and Organizational Psychology and Behavior* 26 113–136. 10.1002/job.270

[B45] Hoyle R. H. Sherrill M. R. (2006). Future orientation in the self-system: possible selves, self-regulation, and behavior. *J. Pers.* 74 1673–1696. 10.1111/j.1467-6494.2006.00424.x 17083662

[B46] Hu L. T. Bentler P. M. (1999). Cutoff criteria for fit indexes in covariance structure analysis: conventional criteria versus new alternatives. *Struct. Equat. Model.* 6 1–55. 10.1080/10705519909540118

[B47] Huppert F. A. (2009). Psychological well-being: evidence regarding its causes and consequences. *Appl. Psychol. – Hlth. We* 1 137–164. 10.1111/j.1758-0854.2009.01008.x

[B48] Iverson R. D. Maguire C. (2000). The relationship between job and life satisfaction: Evidence from a remote mining community. *Hum. Relat.* 53 807–839. 10.1177/0018726700536003

[B49] Jin L. Watkins D. Yuen M. (2009). Personality, career decision self-efficacy and commitment to the career choices process among Chinese graduate students. *J. Vocat. Behav.* 74 47–52. 10.1016/j.jvb.2008.10.002

[B50] Judge T. A. Bretz R. D. Jr. (1994). Political influence behavior and career success. *J. Manage.* 20 43–65. 10.1177/014920639402000103 30868368

[B51] Judge T. A. Klinger R. L. Simon L. S. (2010). Time is on my side: time, general mental ability, human capital, and extrinsic career success. *J. Appl. Psychol.* 95 92–107. 10.1037/a0017594 20085408

[B52] Kahneman D. Diener E. Schwarz N. (eds) (1999). *Well-being: Foundations of hedonic psychology.* Russell: Sage Foundation.

[B53] Kanfer R. Wanberg C. R. Kantrowitz T. (2001). Job search and employment: a personality-motivational analysis and meta-analytic review. *J. Appl. Psychol.* 86 837–855. 10.1037/0021-9010.86.5.837 11596801

[B54] Kihlstrom J. F. Cantor N. (1984). “Mental representations of the self,” in *Advances in experimental social psychology*, Vol. 17 ed. Berkowitz L. (New York: Academic Press), 1–47.

[B55] Kok B. E. Coffey K. A. Cohn M. A. Catalino L. I. Vacharkulksemsuk T. Algoe S. B. (2013). How positive emotions build physical health: perceived positive social connections account for the upward spiral between positive emotions and vagal tone. *Psychol. Sci* 24 1123–1132. 10.1177/0956797612470827 23649562

[B56] Korsgaard M. A. Roberson L. (1995). Procedural justice in performance evaluation—The role of instrumental and noninstrumental voice in performance-appraisal discussions. *J. Manage* 21 657–669. 10.1016/0149-2063(95)90004-7

[B57] Lee Y. T. Reiche B. S. Song D. (2010). How do newcomers fit in? The dynamics between person-environment fit and social capital across cultures. *Int. J. Cross Cul. Manage.* 10 153–174. 10.1177/1470595810370911

[B58] Lent R. W. Brown S. D. Hackett G. (1994). Toward a unifying social cognitive theory of career and academic interest, choice, and performance. *J. Vocat. Behav.* 45 79–122. 10.1006/jvbe.1994.1027

[B59] Lent R. W. Brown S. D. Hackett G. (2000). Contextual supports and barriers to career choice: a social cognitive analysis. *J. Couns. Psychol.* 47 36–49. 10.1037/0022-0167.47.1.36 28836808

[B60] Li M. Wang Z. Gao J. You X. (2017). Proactive personality and job satisfaction: The mediating effects of self-efficacy and work engagement in teachers. *Curr. Psychol.* 36 48–55. 10.1007/s12144-015-9383-1

[B61] Lin Y.-C. Yu C. Yic C. (2014). The effects of positive affect, person-job fit, and well-being on job performance. *Soc. Behav. Personal.* 42 1537–1548. 10.2224/sbp.2014.42.9.1537

[B62] Liu W. Li Z. Ling Y. Cai T. (2016). Core self-evaluations and coping styles as mediators between social support and well-being. *Pers. Indiv. Differ.* 88 35–39. 10.1016/j.paid.2015.08.044

[B63] Locke E. A. (1967). Motivational effects of knowledge of results. *J. Applied Psychol.* 51 324–329. 10.1037/h0026737 6075575

[B64] Locke E. A. Cartledge N. Koeppel J. (1968). Motivational effects of knowledge of results: a goal-setting phenomenon? *Psychol. Bull* 70(6pt1) 474–485.

[B65] Locke E. A. Latham G. P. (2006). New directions in goal-setting theory. *Curr. Dir. Psychol. Sci.* 15 265–268. 10.1111/j.1467-8721.2006.00449.x

[B66] Magee W. (2013). Anxiety, demoralization, and the gender difference in job satisfaction. *Sex Roles.* 695 308–322. 10.1007/s11199-013-0297-9

[B67] Major B. C. Le Nguyen K. D. Lundberg K. B. Fredrickson B. L. (2018). Well-being correlates of perceived positivity resonance: evidence from trait and episode-level assessments. *Personal. Soc. Psychol. Bull.* 44 1631–1647. 10.1177/0146167218771324 29756547 PMC8750237

[B68] Mayrhofer W. Briscoe J. P. Hall D. T. Dickmann M. Dries N. Dysvik A. (2016). Career success across the globe: insights from the 5c project. *Organ. Dyn.* 45 197–205. 10.1016/j.orgdyn.2016.07.005

[B69] Ng T. W. H. Feldman D. C. (2014). Subjective career success: a meta-analytic review. *J. Vocat. Behav.* 85 169–179. 10.1016/j.jvb.2014.06.001

[B70] Pan J. Zhao H. Zhou W. Gong M. (2016). What I have gotten is not what I want — Multi-dimensional analysis on the influence of the differences between career success and the career success evaluation on happiness. *Hum. Resou. Dev. China* 11 6–17. 10.16471/j.cnki.11-2822/c.2016.11.001 (In Chinese),

[B71] Pan J. Zhou W. (2015). How do employees construe their career success: An improved measure of subjective career success. *Int. J. Select. Assess.* 23 45–58. 10.1111/ijsa.12094

[B72] Pan J. Z. Zhou W. X. (2013). Can success lead to happiness? The moderators between career success and happiness. *Asia Pac. J. Hum. Resou.* 51 63–80. 10.1111/j.1744-7941.2012.00033.x

[B73] Peng Y. Mao C. (2015). The impact of person-job fit on job satisfaction: The mediator role of self efficacy. *Soc. Indic. Res.* 121 805–813. 10.1007/s11205-014-0659-x

[B74] Podsakoff P. M. MacKenzie S. B. Lee J. Y. Podsakoff N. P. (2003). Common method biases in behavioral research: a critical review of the literature and recommended remedies. *J. Appl. Psychol.* 88 879. 10.1037/0021-9010.88.5.879 14516251

[B75] Podsakoff P. M. Organ D. W. (1986). Self-reports in organizational research: Problems and prospects. *J. Manage.* 12 531–544. 10.1177/014920638601200408 8452065

[B76] Preacher K. J. Hayes A. F. (2008). *Assessing Mediation in Communication Research.* London: The Sage sourcebook of advanced data analysis methods for communication research, 13–54.

[B77] Ryan R. M. Deci E. L. (2001). On happiness and human potentials: A review of research on hedonic and eudaimonic well-being. *Annu. Rev. Psychol.* 52 141–166. 10.1146/annurev.psych.52.1.141 11148302

[B78] Saks A. M. Ashforth B. E. (1997). A longitudinal investigation of the relationships between job information sources, applicant perceptions of fit, and outwork comes. *Pers. Psychol.* 50 395–426. 10.1111/j.1744-6570.1997.tb00913.x

[B79] Savickas M. L. (2013). Career construction theory and practice. *Career Dev. Couns. Putting theory and research to work.* 2 147–183.

[B80] Seibert S. E. Crant J. M. Kraimer M. L. (1999). Proactive personality and career success. *J. Appl. Psychol.* 84 416. 10.1037/0021-9010.84.3.416 10380421

[B81] Sin N. L. Lyubomirsky S. (2009). Enhancing well-being and alleviating depressive symptoms with positive psychology interventions: a practice-friendly meta-analysis. *J. Clin. Psychol.* 65 467–487. 10.1002/jclp.20593 19301241

[B82] Sortheix F. M. Chow A. Salmanazar K. (2015). Work values and the transition to work life: a longitudinal study. *J. Vocat. Behav.* 2015 162–171. 10.1016/j.jvb.2015.06.001

[B83] Strauss K. Griffin M. A. Parker S. K. (2012). Future work selves: how salient hoped-for identities motivate proactive career behaviors. *J. Applied Psychol.* 97 580–598. 10.1037/a0026423 22122111

[B84] Stumpf S. A. (2014). A longitudinal study of career success, embeddedness, and mobility of early career professionals. *J. Vocat. Behav.* 85 180–190. 10.1016/j.jvb.2014.06.002

[B85] Taber B. J. Blankemeyer M. (2015). Future work self and career adaptability in the prediction of proactive career behaviors. *J. Vocat. Behav.* 86 20–27. 10.1016/j.jvb.2014.10.005

[B86] Tinsley H. E. A. (2000). The congruence myth: an analysis of the efficacy of the person-environment fit model. *J. Vocat. Behav.* 56 147–179. 10.1006/jvbe.1999.1727

[B87] Visagie S. Koekemoer E. (2014). What it means to succeed: personal perceptions of career success held by senior managers. *S. Afr. J. Bus. Manag.* 45 43–54. 10.4102/sajbm.v45i1.116

[B88] Wang Q. Bowling N. A. Eschleman K. J. (2010). A meta-analytic examination of work and general locus of control. *J. Applied Psychol.* 95 761. 10.1037/a0017707 20604595

[B89] Waterman A. S. (1993). Two conceptions of happiness: contrasts of personal expressiveness (eudaimonia) and hedonic enjoyment. *J. Pers. Soc. Psychol.* 64 678–691. 10.1037/0022-3514.64.4.678

[B90] Watson D. Clark L. A. Tellegen A. (1988). Development and validation of brief measures of positive and negative affect: the PANAS scales. *J. Pers. Soc. Psychol.* 54 1063. 3397865 10.1037//0022-3514.54.6.1063

[B91] Xin L. Zhou W. Tang F. (2019). Antecedents of career success criteria clarity: based on social cognitive career theory. *Business Management Journal* 4 127–141. 10.19616/j.cnki.bmj.2019.04.008

[B92] Zhou W. Guan Y. Xin L. Mak M. C. K. Deng Y. (2016). Career success criteria and locus of control as indicators of adaptive readiness in the career adaptation model. *J. Vocat. Behav.* 94 124–130. 10.1016/j.jvb.2016.02.015

[B93] Zhou W. Sun J. Guan Y. Li Y. Pan J. (2013). Criteria of career success among Chinese employees: developing a multi-dimensional scale with qualitative and quantitative approaches. *J. Career Assess.* 21 265–277. 10.1177/1069072712471302

